# Discovery and annotation of a novel transposable element family in *Gossypium*

**DOI:** 10.1186/s12870-018-1519-7

**Published:** 2018-11-28

**Authors:** Hejun Lu, Xinglei Cui, Zhen Liu, Yuling Liu, Xingxing Wang, Zhongli Zhou, Xiaoyan Cai, Zhenmei Zhang, Xinlei Guo, Jinping Hua, Zhiying Ma, Xiyin Wang, Jinfa Zhang, Hong Zhang, Fang Liu, Kunbo Wang

**Affiliations:** 1 State Key Laboratory of Cotton Biology, Institute of Cotton Research of Chinese Academy of Agricultural Science, Anyang, 455000 Henan China; 2 Gembloux Agro-Bio Tech, University of Liège, 5030 Gembloux, Belgium; 3 Anyang Institute of Technology, Anyang, 455000 Henan China; 4 Beijing Key Laboratory of Crop Genetic Improvement, China Agricultural University, Beijing, 100193 China; 5 Key Laboratory for Crop Germplasm Resources of Hebei province, Hebei Agricultural University, Baoding, 071000 Hebei China; 6 Center for Genomics and Computational Biology, North China University of Science and Technology, Tangshan, 063000 Hebei China; 7 Department of Plant and Environmental Sciences, New Mexico State University, Las Cruces, 88003 USA; 8 Department of Biological Sciences, Texas Tech University, Lubbock, 79409 USA

**Keywords:** FISH, *Ty3/Gypsy*, *Gossypium*, Transposable element, Allotetraploid, Evolution

## Abstract

**Background:**

Fluorescence *in situ* hybridization (FISH) is an efficient cytogenetic technology to study chromosome structure. Transposable element (TE) is an important component in eukaryotic genomes and can provide insights in the structure and evolution of eukaryotic genomes.

**Results:**

A FISH probe derived from bacterial artificial chromosome (BAC) clone 299N22 generated striking signals on all 26 chromosomes of the cotton diploid A genome (AA, 2x=26) but very few on the diploid D genome (DD, 2x=26). All 26 chromosomes of the A sub genome (At) of tetraploid cotton (AADD, 2n=4x=52) also gave positive signals with this FISH probe, whereas very few signals were observed on the D sub genome (Dt). Sequencing and annotation of BAC clone 299N22, revealed a novel Ty3/gypsy transposon family, which was named as ‘*CICR’*. This family is a significant contributor to size expansion in the A (sub) genome but not in the D (sub) genome. Further FISH analysis with the LTR of *CICR* as a probe revealed that *CICR* is lineage-specific, since massive repeats were found in A and B genomic groups, but not in C–G genomic groups within the *Gossypium* genus. Molecular evolutionary analysis of *CICR* suggested that tetraploid cottons evolved after silence of the transposon family 1–1.5 million years ago (Mya). Furthermore, A genomes are more homologous with B genomes, and the C, E, F, and G genomes likely diverged from a common ancestor prior to 3.5–4 Mya, the time when *CICR* appeared. The genomic variation caused by the insertion of *CICR* in the A (sub) genome may have played an important role in the speciation of organisms with A genomes.

**Conclusions:**

The *CICR* family is highly repetitive in A and B genomes of *Gossypium*, but not amplified in the C–G genomes. The differential amount of *CICR* family in At and Dt will aid in partitioning sub genome sequences for chromosome assemblies during tetraploid genome sequencing and will act as a method for assessing the accuracy of tetraploid genomes by looking at the proportion of *CICR* elements in resulting pseudochromosome sequences. The timeline of the expansion of *CICR* family provides a new reference for cotton evolutionary analysis, while the impact on gene function caused by the insertion of *CICR* elements will be a target for further analysis of investigating phenotypic differences between A genome and D genome species.

**Electronic supplementary material:**

The online version of this article (10.1186/s12870-018-1519-7) contains supplementary material, which is available to authorized users.

## Background

The C-value paradox is a term used to describe the finding that the amount of organismal DNA does not correlate linearly with the number of functional genes. This paradox is not only restricted to distantly related organisms, but also observed among closely related species [1]. The amplification and proliferation of repetitive sequences, especially transposable elements (TEs), is the main reason for the variation of genome size among organisms. Repetitive DNA accounts for a huge fraction of the genome in most organisms [2, 3], and TEs are thought to have played important roles in such as, variations in intron size [4], segmental duplication [5, 6], transfer of organelle DNA to the nucleus [7], expansion/contraction of tandem repeats and illegitimate recombination [8], which all contribute to the C-value paradox [9–11]. Long Terminal Repeat Retrotransposons (LTR-RTs), which are usually scattered throughout genomes, are the most abundant TE type, and often cause genome expansion; this is particularly the case for plants. LTR-RTs can spread rapidly throughout their host genomes, leading to significant increase in genome size over a short evolutionary period [12]. For example, in a span of just few million years, 70% of the maize genome was composed of LTR-RTs, and its size had increased two- to five-fold due to TE activity [13]. *Oryza australiensis*, also had a rapid two-fold increase in its genome size due to a recent burst of three LTR-RT families during the last three million years [12]. The specific proliferative pattern of families and classes of dispersed repetitive elements can vary widely, even between closely related *Gossypium* lineages [10]. LTR-RTs, which are ubiquitous and highly abundant in plant genomes, also account for a large fraction in *Gossypium* genomes [14–20].

The genus *Gossypium* includes over 50 recognized species that are divided into diploid and tetraploid lineages [21]. The former lineage includes 45 species (2n =2x = 26), which are further classified into eight genomes (A–G, and K); this classification is mainly based on chromosome pairing behaviors and the fertility of interspecific hybrids [22, 23]. Tetraploid cottons, which include two domesticated species, *G. hirsutum* and *G. barbadense,* and four recognized polyploid species, *G. tomentosum*, *G. mustelinum*, *G. darwinii*, and *G. ekmanianum* [24] were believed to be the product of hybridization between two parental diploid species with A and D genomes [23]. The genome size of diploid members ranges approximately threefold, from 1.81 pg to 5.26 pg [22], which provides a model system for studying genome-size variation.

Fluorescence *in situ* hybridization of bacterial artificial chromosome (BAC-FISH) can locate BAC clones with different characteristics directly to chromosomes. It is used widely in plant molecular cytogenetic studies such as karyotyping, gene mapping, chromosome identification and physical mapping [25]. With the BAC-FISH approach, major components from biased hybridization have recently been characterized, leading to the demonstration of allotetraploidy in the ginseng genome [26]. Also using BAC-FISH, Liu and colleagues reported a repeats-enriched cytogenetic marker for distinguishing cotton A and D genomes, and a Gypsy-LTR-RT in heterochromatic regions, which was thought as a reason to cause the size variation between A and D genomes [27].

In this study, we reported a peculiar BAC 299N22 screened from the *G. barbadense* BAC library, which exhibited striking biased hybridization signals between diploid A and D genomes, as well as between At and Dt in tetraploids. The sequencing of BAC clone 299N22 leaded to the identification of a novel LTR, which was subsequently found to be responsible for the biased hybridization signals of the BAC clone. The LTR was belonged to a specific Ty3/gypsy family that accounts for a considerable proportion of the A (sub) genome, but is completely absent in the D (sub) genome. The distribution and evolution analyses of this family in the representative species of each genomic group of cotton provided further insights into the speciation of *Gossypium* genus. Analysis of the insertion of this LTR-RT family into genes of the A genome will prove a new approach for revealing trait differences between *Gossypium* species.

## Methods

### Plant materials and BAC library

Twenty cotton species were used in this study, including 5 tetraploids (2n=4x=52) and 15 diploids (2n=2x=26). The genomes and accession names of the species are (1) *G. hirsutum*, (AD)_1_, CCRI-12; (2) *G. barbadense*, (AD)_2_, Xinhai-7; (3) *G. tomentosum*, (AD)_3_, (AD)3-11; (4) *G. mustelinum*, (AD)_4_, (AD)4-16; (5) *G. darwinii*, (AD)_5_, (AD)5-7; (6) *G. herbaceum*, A_1_, Hongxingcaomian; (7) *G. arboreum*, A_2_, SHIXIYA-1; (8) *G. herbaceum subs africanum*, A_1-a_, A1a00; (9) *G. anomalum*, B_1_, B1-9; (10) *G. captis-viridis*, B_3_, B3-1; (11) *G. sturtianum*, C_1_, C1-4; (12) *G. thurberi*, D_1_, D1-5; (13) *G. davidsonii*, D_3-d_, D3d-1; (14) *G. aridum*, D_4_, D4-1; (15) *G. raimondii*, D_5_, D5-7; (16) *G. gossypioides*, D_6_, D6-6; (17) *G. trilobum*, D_8_, D8-5; (18) *G. stocksii*, E_1_, E1-00; (19) *G. longicalyx*, F_1_, F1-3; (20) *G. austral*, G_2_, G2-1. All the plant material was grown perennially at National Wild Cotton Nursery in Sanya city, Hainan Island, China, which is supervised by the Institute of Cotton Research of Chinese Academy of Agricultural Sciences located in Anyang City, Henan Province, China.

The Pima 90-53 BAC library that was screened in this paper was provided by Prof. Zhiying Ma (Hebei Agricultural University, China).

### Genome sequence data

*G. raimondii* genome assembly was downloaded from the sequenced genome at Phytozome (https://phytozome.jgi.doe.gov/pz/portal.html) [20]. *G. arboreum* genome sequence was downloaded from Cottongen (https://www.cottongen.org/) [18]. Different versions of *G. hirsutum* and *G. barbadense* genomes, and the gff file and Gene ontology (GO) annotation file of *G. hirsutum* used for GO analysis were obtained from Cottongen (https://www.cottongen.org/).

If not specified, the genome data, TE proportions, and gff information of *G. raimondii*, *G. hirsutum*, *G. barbadense* referred to in the analyses in this study were from three sources [14, 16, 20].

### BAC clone 299N22 sequencing and annotation

Sequencing and assembly of BAC clone 299N22 using Ion Torrent PGM technology was outsourced to Shanghai Invitrogen Inc. After the sequencing, two scaffolds of BAC clone 299N22 were obtained and submitted into NCBI, with accession of MH713613 (Scaf 01) and MH713614 (Scaf 02). Online CD-search was performed to search for coding genes (https://www.ncbi.nlm.nih.gov/Structure/cdd/wrpsb.cgi) [28]. The online program CENSOR (http://www.girinst.org/)[29] was used to search known repeats from the Repbase database [30].

### FISH

Mid-mitotic chromosomes were selected for FISH, with the exception of *G. longicalyx* (F_1_), for which meiosis pachytene chromosomes were used as target DNA. The probes were labeled according to the instructions of the manufacturer (Roche Diagnostics, USA).

Chromosome preparation and the FISH procedure were conducted using modifications of previous protocols [31–33]. The PCR products of paired primers (left: CGGCACCAAAAACTTGCTATGT, right: GATGTTATACGGGGTGTGCCG) designed with the template of the left LTR of *CICR_Ga001* were used as probe to do FISH experiment. The amplification procedure was: firstly, 95 °C 5 min for predegeneration; then 95 °C for 30 s, 56 °C for 30 s, 72 °C min for 1.5 min, 30 cycles; the final extension is at 72 °C for 6 min. The composition of the reaction mix using the following: gDNA (~5 μg/ml), primers (~0.8 μM), PCR Master Mix (Thermo), and H_2_O.

### Transposon structure, genome proportions, and expansion time

MGEScan_LTR (http://darwin.informatics.indiana.edu/cgi-bin/evolution/daphnia_ltr.pl) software was used to search for LTRs. Web LTR_FINDER was used to accurately predict the locations and structure of full-length LTR retrotransposons by considering common structural features [34]. Individual LTR-RTs were classified with the 80-80 rule: if two sequences share more than 80% of their coding or internal domains, or within their terminal repeat regions, or in both, the two sequences can be classified into same family [35]. The insert enzymes were annotated by using the online CD-search. RepeatMasker was used to estimate the proportion of TEs in the *Gossypium* genome, and the estimated proportion of *CICR* was then obtained by using an in-house Perl script to remove redundancy. We aligned the 5′- and 3′-ends of the LTR sequences of each retrotransposon using MUSCLE [36], and the distances were calculated based on the Jukes-Cantor formula using the distmat program of EMBOSS toolkit [37]. The divergence time of the LTR was estimated using the formula T = d/2r, where r represents a synonymous substitution rate of 1.3 × 10^−8^ per site per year [38].

### Collinearity, TE and gene distribution, GO annotation

The multiple micro-collinearity of homologous regions in chromosome 13 of At and Dt were performed by in-house Perl scripts based on the BLASTN result (Version 2.6.0) [39]. The LTR assignments and gene distributions along chromosomes were made by Circos [40], the gene locations were obtained from a coding sequence (CDS) gff annotation file contained in the *G. hirsutum* genome sequencing program [14]. GO results were illustrated using BGI WEGO (http://wego.genomics.org.cn/) [41].

## Results

### FISH, sequencing and annotation of BAC clone 299N22

The clone 299N22 from our previous research [42], showed strong hybridization signals distributed on all A (sub) genome chromosomes, but were almost absent in D (sub) genome. The differential FISH signals between A (sub) genome and D (sub) genome chromosomes prompted us to sequence this clone. Two scaffolds, which are separated by an interval of simple repeat sequences, were obtained with sizes of 6,652 bp (Scaf 01) and 93,071 bp (Scaf 02). BLASTN with cotton genome sequences was performed by using the two scaffolds as query sequences. Matches were detected in D_5_13 chromosome at the ~58.4% region, which was consistent with the relative position of FISH signals, and could explain the single pair of BAC-FISH signals on D_5_13 chromosome [42]. On the other hand, a segment with length of ~1.4 kb from Scaf 02, which had thousands of matched copies in A_2_ genome (Additional file 3: Table S1), was identified. The ~1.4 kb length sequence may explain the dispersed BAC-FISH signals on all chromosomes in the A_2_ genome.

Conserved domain search was performed to identify possible genes contained in the 1.4 kb segment [28]. No conserved domain was found, indicating that this segment likely does not contain any genes. CENSOR was performed to screen the 1.4 kb segment against reference collection of repeats found in eukaryotic genomes (https://www.girinst.org/repbase/), no match was found, indicating that the segment contains a novel repeat element.

By MGEScan_LTR, the 1.4 kb segment was identified as a novel LTR, and one kind of TE with two ends of this LTR were discovered in *G. arboreum*. To mine this type of TE in different cotton genomes, we ran RepeatMasker against the whole genome sequences of *G. arboreum* [18], *G. raimondii* [20], *G. hirsutum* [14], and *G. barbadense* [16] with this novel LTR-RT as query sequence. Results showed there are abundant repeats of the TE both in A_2_ and At genomes (Table 1, Additional file 4: Table S2), but complete absence in D_5_ and Dt genomes, confirming that these LTR-RTs are lineage-specific among *Gossypium* species. As the investigated genome sequences were produced with short read technologies, it is likely that the amount of the highly repetitive LTR-RT may not be accurately estimated, however the results present basic coincidence with our FISH experiments.

**Table 1:** The proportion of *CICR* in A_2_ and At

Category	A_2_ (Mb)	A_2_ (%)	GhA_t_ (Mb)	GhA_t_ (%)	GbA_t_ (Mb)	GbA_t_ (%)
A (At)	1561.32	100	1220.73	100	1394.66	100
TE	966.30	61.89	843.52	69.11	905.13	64.9
LTR	700.97	42.98	625.38	51.23	384.98	27.60
Gypsy	397.67	25.47	361.96	29.65	209.19	15
*CICR*	193.82	12.41	165.54	13.56	57	4.09
*CICR*-LTR*	4931		4862		4924	
Intact CICR*	113		140		9	

*CICR*-LTR* means the number of *CICR*-LTR consisted in the whole genome or sub-genome. Intact CICR* means the number of *CICR* members

**Fig. 1:**
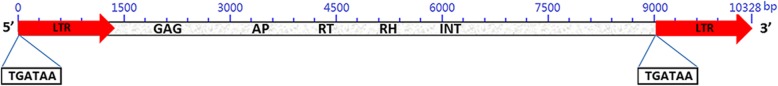
Graphic representation of the structural features identified from *RLG_CICR_Ga001* drawn to scale. GAG: capsid protein; AP: aspartic proteinase; INT: integrase; RT: reverse transcriptase; RH: RNase H

The structure of one intact TE was characterized (Fig. 1), revealing that these TEs belong to the super-family of Gypsy/Ty3 according to the TE nomenclature [35]. We named the TEs as *RLG_CICR* based on the classification system and nomenclature, where *CICR* represents *Chinese Institute of Cotton Research* (Institute of Cotton Research, Chinese Academy of Agricultural Sciences). Intact *CICR* family members had a mean size of 10221 bp, with a mean LTR size of 1362 bp starting with 5´-TG-3´ and ending with 5´-CA-3´ (Additional file 1: Figure S1). They produced target site duplication (TSD) of TGATAA, and typically contained conserved domain for GAG, AP, RT, RH, and INT within their sequence (Fig. 1).

### FISH recurrence and broadening of *CICR* probe reactivity to other *Gossypium* species

**Fig. 2:**
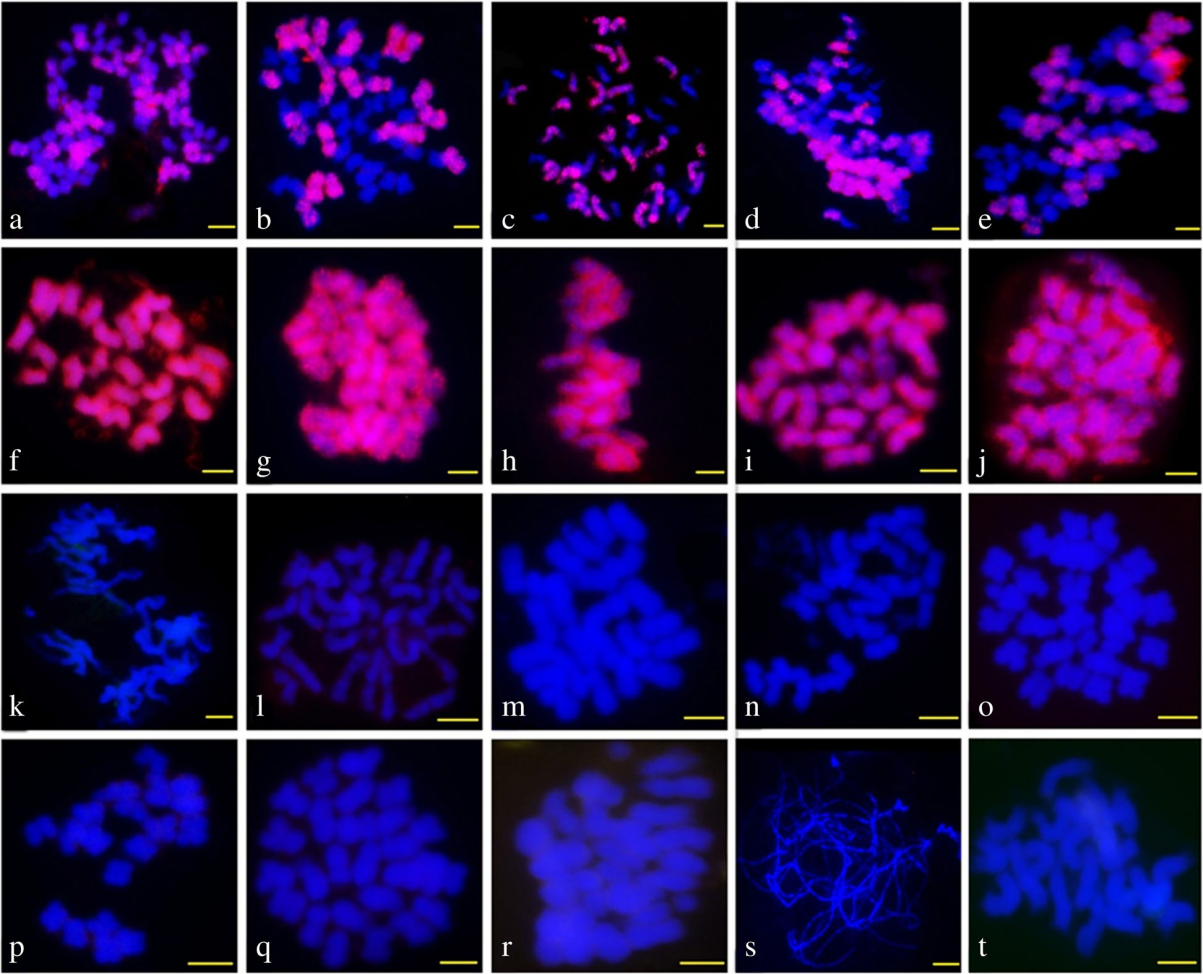
The FISH images of LTR sequence (red) hybridized to mitotic chromosomes (meiosis pachytene chromosomes of *G. longicalyx*) of 20 *Gossypium* species, **a**: *G. hirsutum* ((AD)_1_); **b**: *G. barbadense* ((AD)_2_); **c**: *G. tomentosum* ((AD)_3_); **d**: *G. mustelinum* ((AD)_4_); **e**: *G. darwinii* ((AD)_5_); **f**: *G. herbaceum* (A_1_); g: *G. arboreum* (A_2_); **h**: *G. herbaceum subs africanum* (A_1_); **i**: *G. anomalum* (B_1_); **j**: *G. capitis-viridis* (B_3_); **k**: *G. sturtianum* (C_1_); **l**: *G. thurberi* (D_1_); **m**: *G. davidsonii* (D_3-d_); **n**: *G. aridum* (D_4_); **o**: *G. raimondii* (D_5_); **p**: *G. gossypioides* (D_6_); **q**: *G. trilobum* (D_8_); **r**: *G. stocksii* (E_1_); **s**: *G. longicalyx* (F_1_); **t**: *G. austral* (G_2_). Bar=5 μm

The next key step was to explore whether *CICR* can recur the FISH signals. The PCR product from the *CICR* family (the left LTR of *RLG_CICR_Ga001*) were applied as probe to FISH on mitotic chromosomes of the two A genome (A_1_ & A_2_), two D genome (D_5_ & D_1_) and five tetraploid cotton species ((AD)_1_, (AD)_2_, (AD)_3_, (AD)_4_ & (AD)_5_). A similar FISH Signals to that exhibited by BAC-FISH was observed, strong hybridization signals in A and At genomes, while no signal on D & Dt genomes (Fig. 2). The results revealed that *CICR*-LTRs were responsible for the biased hybridization signals of BAC clone 299N22 between A and D genomes.

For broader evaluating the distribution of *CICR* family in other *Gossypium* genomes, more cotton species were used, including one A (A_1-a_), four D (D_3-d_, D_4_, D_6_ & D_8,_), two B (B_1_ & B_3_) and one representative of diploid C, E, F and G genomes (C_14_, E_14_, F_1_ & G_2;_ no K genomes were tested due to a lack of suitable material for the FISH analysis). The LTR-FISH signals were distributed in all chromosomes of B genome species, but were less widespread in C, E, F, and G genomes (Fig. 2).

### Contribution of *CICR* in A genome size expansion

According to BLASTN results, around 4,900 *CICR*-LTRs were identified scattered on 13 pairs of A (At) chromosomes, and 113, 140 and 35 intact *CICR* members were identified in *G. arboreum* [18], *G. hirsutum* (At) [14] and *G. barbadense* (At) [16], respectively (Table 1, Additional file 4: Table S2). According to the RepeatMasker results, the *CICR* family accounts for 12.41%, 13.56%, and 4.09% of the genome size in A_2_, GhAt, and GbAt, respectively. The complete absence of this LTR-RT family in the D genome partially explains the difference in size between A and D genomes. For example, about 23% of the 830 Mb difference in genome size between *G. arboreum* and *G. raimondii* (1.56 Gb: 0.73 Gb) [17, 20] can be explained by *CICR* dynamics.

**Fig. 3:**
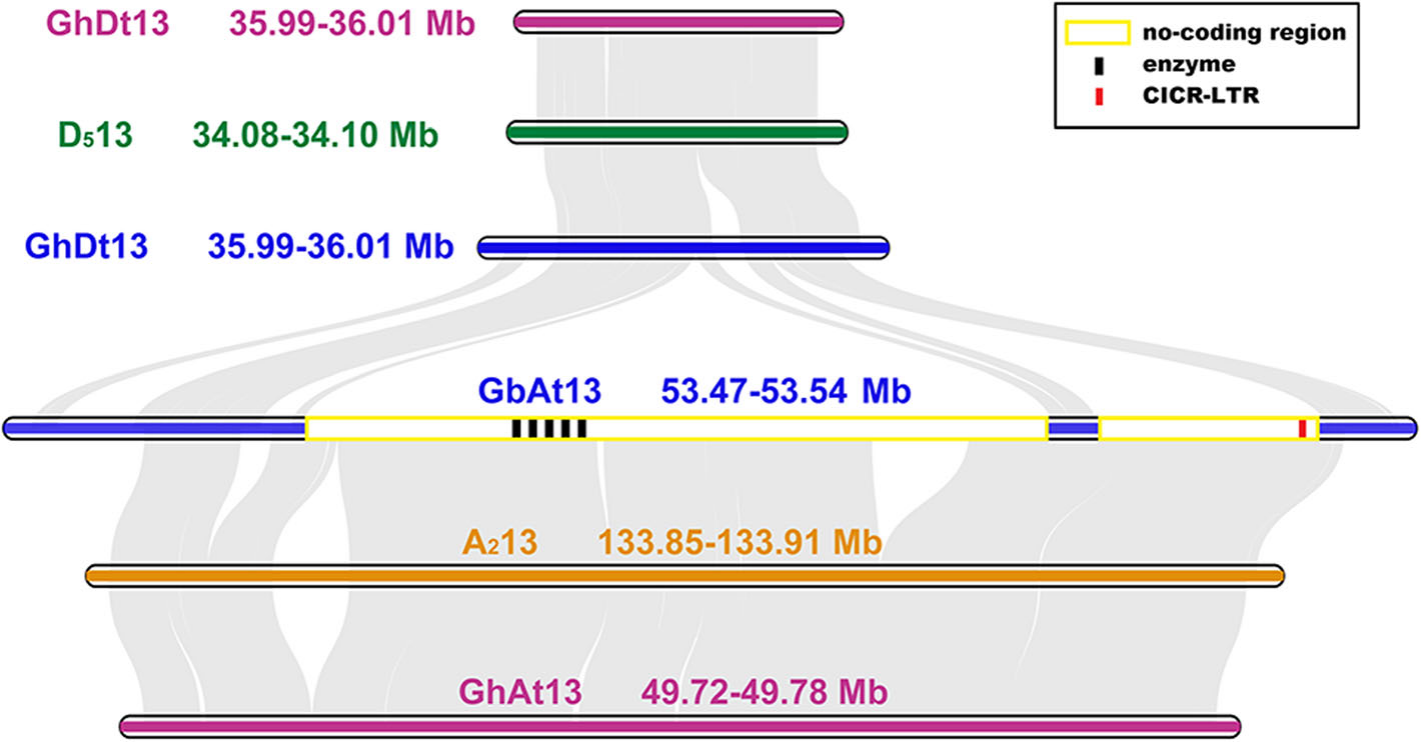
Micro-collinearity between homologous fragments extracted from A_2_13, D_5_13, Dt13, At13. The rectangular box with yellow border in GbAt13 represent the insert region, where the black strips represent GAG, AP, RT, RH, and INT from left to right, and the red strip represent *CICR*-LTR, the other white region represent the no-coding regions

To examine the genomic changes caused by the insertion of *CICR* members, the micro–collinearity between homologous regions of the BAC clone 299N22 in different genomes were presented as one example (Fig. 3). Homologous regions in A_2_ and D_5_ genomes, and in At and Dt genomes of *G. hirsutum* and *G. barbadense* were extracted based on the BLAST results of homologous genes and common Simple sequence repeats (SSR) markers. Discrepancies among A and At genomes were more frequent than between D and Dt, indicating that A and At have accumulated more mutations during evolution, while D and Dt remained more conserved. The size of homologous regions from the A (sub) genome was 3 times of that extracted in the D (sub) genome, mainly due to the insertion of large non-coding regions accompanied by five transposon-related enzymes and one *CICR*-LTR inside. Thus, the size enlargement and greater variation in the A (sub) genome may be caused by the insertion of *CICR*.

### Amplification of the *CICR* family and speciation time in *Gossypium*

**Fig. 4:**
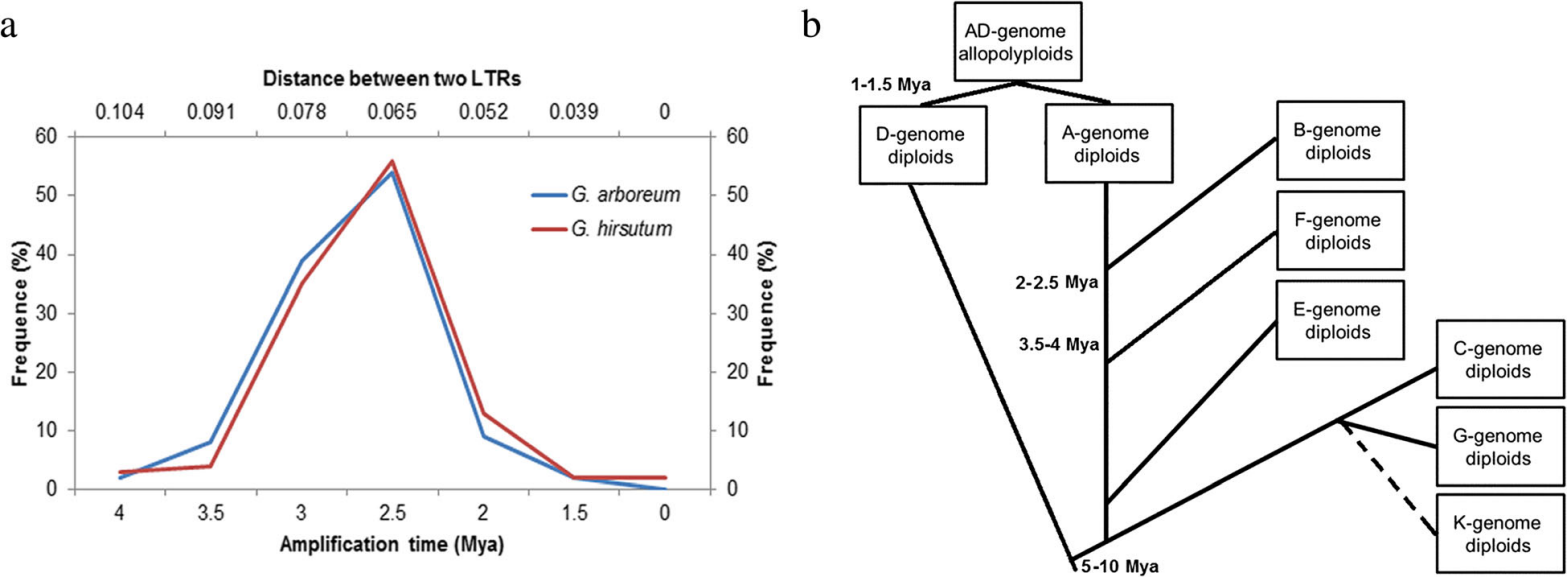
The Amplification time of *CICR* in *G. arboreum* and GhAt (**a**), and the phylogeny tree of *Gossypium* species (**b**)

To study *CICR* evolution*,* we applied genomic paleontology, an approach consisting of sequence divergence translating between left and right LTRs of all intact members into a kind of radiation data which relies on a base substitution rate of 1.3×10^-8^, referred to as “r” [38]. *CICR* in *G. hirsutum* had a strikingly similar pattern to those in *G. arboreum* on the trend curve (Fig. 4a), while *CICR* in *G. barbadense* were not included here due to the scarceness of intact elements. The pairwise distances (d) of each pair of left and right LTRs ranged from 0–0.104, which indicated that the elements amplified within the last 4 million years (Mya), peaked within 2.5 Mya (Fig. 4a). Furthermore, the data showed that transpositional activity was sporadic over the last 1.5 Mya. The dates of *CICR* point to a recent sudden burst in retrotransposon activity that played a major role in the enlargement of cotton genome.

**Fig. 5:**
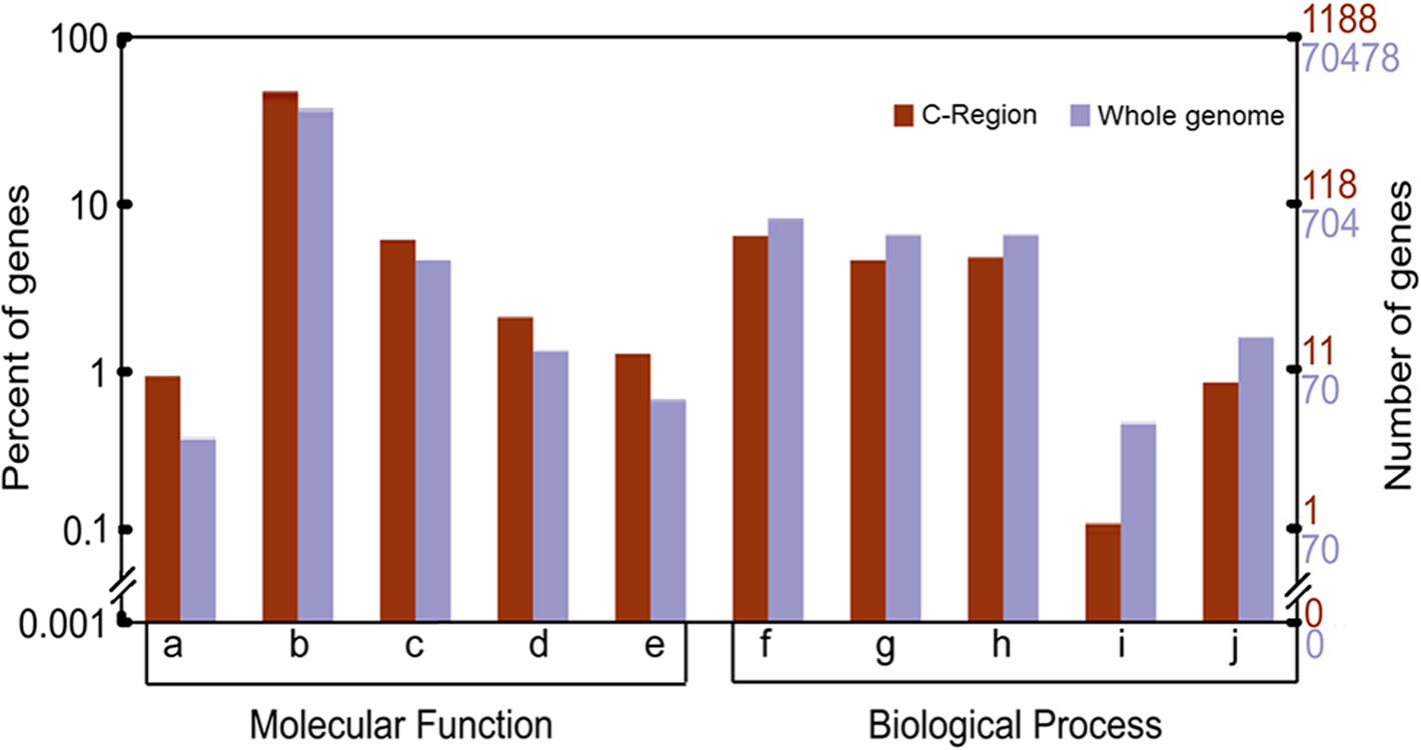
The difference of GO cluster between gene set located in C-Region and the whole genome. The ordinate take log10 of numerical value. GO annotation, a: carbohydrate binding, b: catalytic, c: oxidoreductase, d: oxidoreductase, acting with incorporation or reduction of molecular oxygen, e: transferase, transferring one-carbon groups, f: biosynthetic process, g: cellular biosynthetic process, h: gene expression, i: macromolecule biosynthetic process, j: response to chemical stimulus

According to the distribution of *CICR* in different genomes as revealed by FISH (Fig. 2), we were able to infer the evolution of the *Gossypium* species. The divergence of A and D diploids should have occurred at least 4 Mya ago (i.e., before the appearance of *CICR*) so that *CICR* was active in A but not D, which is in accordance with a previous study [14, 43]. The origin of the AD allotetraploid was circa 1–2 Mya after the silencing of *CICR*, so *CICR* did not move to the Dt from the tetraploid At. The divergences of C, E, F, G genomes from the ramifying line is likely before 3.5-4 Mya, the time at which *CICR* appeared. A and B genomes likely diverged after 2.5 Mya, when the amplification of *CICR* reached a peak, since many TE repeats were detected in the B genome (Fig. 4b). We suggest that the A and B genomes are the most homologous among those in the *Gossypium* genus, which is in contrast to earlier reports that the F genome is more closely related to A than B genome [44].

### *CICR* insertion, and genomic and genetic variation in *Gossypium*

Amplification of TEs will inevitably lead to changes in genomic structure and even gene variation [9]. We graphically displayed the physical locations of genes and *CICR* members in the whole GhAt genome (Additional file 2: Figure S2). In *G. hirsutum*, the genes tend to be centrally distributed at the chromosome ends, while the *CICR*-LTRs were densely distributed in the proximal end region, and were sparsely distributed in the end regions of chromosomes. There were no obvious exclusionary regions between genes and *CICR* members; their overall distributions blended, which is inconsistent with a previous report that LTR-RTs are increasingly dense toward the heterochromatic pericentromeric regions [45].

Structural analysis of all 28749 genes annotated in the chromosomes of GhAt revealed that eighteen genes had insertions of *CICR*-LTR that manifested as introns (Additional file 4: Table S2). Searching with BLASTN in GhDt revealed that fifteen *CICR*-LTR-insert genes pair with their homologous genes in Dt, and all the genes of At are longer than their Dt homologs in total gene size. Another three genes were classified as At-specific, as no homologs were found in Dt; these genes may have been created by the *CICR* activity. Of the fifteen paired homologous genes, ten At genes were highly similar in CDS region to their homologs in Dt, and *CICR*-LTR acted as intron in these genes, and accounted for increased gene size. By contrast, five of the paired homologous genes showed considerable variety between At and Dt. Specifically, this was due to differences in exon size, and to low matching similarity. In general, transposon insertion can lead to genetic structural mutations (increased intron size), affect gene expression (exon changes), and facilitate the creation of new genes.

Some *CICR* members clustered in discrete chromosome regions (Additional file 2: Figure S2). We selected 15 so-called ‘*CICR* Regions’ (C-Regions) here, which harbor at least two *CICR* members with an interval region less than 3Mb. In the C-regions, we analyzed the function of the gene adjacent to *CICR* in an attempt to reveal the functional interaction of gene with *CICR*. A total of 1188 genes were identified in the C-regions, and were classified based on GO annotation. Comparative analysis of the genes functional accumulation between the C-Region and the whole genome (1188/70478), indicated in C-Region five items related to molecular function increased, and another five items involved in biological processes were down regulated (Fig. 5). The increased genes participating in carbohydrate binding, catalysis, oxidation-reduction, and transfer, likely contribute to the promotion of TE packaging and activity. The reason for suppression of biosynthetic processes, gene expression, and response to chemical stimulus remain unclear. The selective accumulation and reduction of gene function in the C-regions suggests that *CICR* insertion should interact with or impact the nearby genes.

## Discussion

### Annotation of a novel TE family in *Gossypium*

Our research stemmed from the discovery of BAC clone 299N22, which showed widespread FISH signals in all the chromosomes of *Gossypium* A (sub) genome, but almost absent in the D (sub) genome [42]. By sequencing the BAC clone, one novel Ty3/*gypsy* TE family was mined from the A (sub) genome. Since it had not been annotated before, we named it ‘*CICR’*. This TE family was characterized with respect to its intact structure, proportion of genomic size, activity date and impact on genome variation. As its special existence in A and B genetic groups of *Gossypium*, the *CICR* family also provides a unique resource for study on the speciation and evolution of *Gossypium*.

### *Gossypium* evolutionary history

In the evolution analysis of LTR transposons, the variability between the 5´- and 3´-LTRs of each retrotransposon has often been used as a measurement of the evolution time of transposons [15]. In this study, the determination of the intact structure of *CICR*s provided materials for calculation of the transposon-active date.

Most expansions of extant LTR retrotransposons occurred independently after lineage separation, but before allotetraploidization [15]. The analysis of *CICR* LTRs indicated that A and D genomes most likely diverged at least 4 Mya. This is coincident with previous reports that the divergence time between the A and D progenitor genomes was ~5–10 Mya [14, 16, 43, 46]. While Li and his colleagues suggested the divergence time for *G. arboreum* and *G. raimondii* is 2–13 Mya [18]. Most previous reports suggested that tetraploids are classic natural allotetraploids that originated in the New World approximately 1–2 Mya; this was a result of hybridization between an A genome ancestral and a D genome species [23, 43, 46, 47]. Recently Li and colleagues showed polyploidization events were predicted to have occurred ~1.5 Mya [17] and Zhang and colleagues pointed out that allotetraploids formed ~1–1.5 Mya [14]. Here, the consistent FISH *CICR* signal between sub genomes of tetraploid and diploid A and D genomes, supported recent experimental evidence for an allopolyploidization event that involved a D genome and an A genome diploid species as parents [48]. Moreover, allotetraploid cotton is suggested to have been formed after silencing of the TE family 1–1.5 Mya, since *CICR* is retained in At but does not transferred to Dt.

Molecular data [46] uniformly supports the recognition of the A, B, E, and F genomic groups as one ramifying line. However, Grover and colleagues suggest that A and F genome are most homologous [44]. Since we detected a high density of *CICR*-FISH signals in diploid A and B genomes, we suggest that they are more closely related to each other than either is to the F genome. However, more molecular evolution evidence is needed to fully support our claim. In summary, we have delineated a new *Gossypium* species phylogenetic tree with time nodes.

### Identification of homologous chromosomes in tetraploids

It is challenging to assemble homologous fragments from sub genome in genome sequencing programs [48]. So *CICR*, which belongs specifically to At, can be an efficient reference to homologous scaffolds that are located between At and Dt in tetraploids. We inspected the distribution of *CICR-*LTR in two versions of the *G. hirsutum* and *G. barbadense* genome assemblies respectively, for convenience here, which were termed as (AD)_1_-NBI [14], (AD)_1_-BGI [17], (AD)_2_-CAS [16], (AD)_2_-HAU [15] (Table 2, Additional file 6: Table S4). The *CICR-*LTRs were queried in all Dt chromosomes of *G. hirsutum* assembly (AD)_1_-BGI, while in the other three tetraploid assemblies, *CICR-*LTR were only queried in At chromosomes, which consistent with the our FISH observations in section 3.1. These comparison revealed the (AD)_1_-BGI perhaps contain much miss assembling between sub genome homologous segments. Towards *G. barbadense*, the (AD)_2_-CAS harbored 4924 *CICR*-LTRs, obviously more than the (AD)_2_-HAU (1049), but consistent in level with *G. arboreum* (4931) and *G. hirsutum* (4862). Thus, *CICR* as one sub genome specific marker can be used to value the accuracy of tetraploid assembly and guide the correct assembling of homologs segments.

**Table 2:** The distribution of *CICR*_LTR in different genome assemblies of tetraploid cotton

Assemblies	Distribution of *CICR*_LTR
(AD)_1_-NBI	A_h_01-A_h_13; None in D-sub genome
(AD)_1_-BGI	A_h_01-A_h_13; D_h_01-D_h_13
(AD)_2_-CAS	A_b_01-A_b_13; None in D-sub genome
(AD)_2_-HAU	A_b_01-A_b_13; None in D-sub genome

Compared with A_2_ and GhAt, GbAt contains less intact *CICR* members in the whole genome, although the numbers of LTR repeats of *CICR* were almost equivalent (Table 1). To ensure this was not due to mistakes in genome assembly, we also examined another *G. barbadense *[15], and obtained consistent results (Additional file 5: Table S3). This maybe because more interruptions have occurred in *CICR* in *G. barbadense*.

### A path for studying phenotypic difference

TEs were recognized as a constantly changing and rich pool of genetic and epigenetic variation where selection can operate, because TE activity would cause a vast range of changes in gene function and expression [9, 49]. Moreover, MITEs (miniature inverted-repeat transposable elements) were reported to regulate the expression of nearby genes [50–52]. In addition, a homeodomain-leucine zipper gene inserted with a copia-like retrotransposon is linked to the hairless phenotype in stem of cotton [53]. Additional research highlights the important role played by introns with regard to gene expression [54, 55]. A_2_ and D_5_ evolved from the same ancestor, but have substantial agronomic differences, since A_2_ genome plants produce textile fiber, whereas and D_5_ genome plants do not [48]. *CICR* played a major role in A genome expansion and constitutes a considerable proportion of the genome. From this feature of *CICR,* we speculate that *CICR* should have also influenced *Gossypium* phenotype. Moreover, we observed the insertion of *CICR* in genes as intron regions and the accumulation of special genes in C-regions. All the findings indicated that *CICR* may have been a critical determinant of the speciation of A genome species. Our annotation of *CICR* provides the research community with a new conceptual framework upon which to base further studies of *Gossypium* speciation and phenotypic differences.

## Conclusions

A novel TE family *CICR* that is lineage specific in *Gossypium* genomes was annotated in this study. The *CICR* family is highly repetitive in the A and B genomes, but almost absent in the C–G genomes. The difference of *CICR* family in cotton genomes showed that the family is an important reason for the genome variation. The annotation of *CICR* family can also aid in genome sequencing and act as methods for assessing accuracy of genome assemblies. The activity of *CICR* family provides a new reference for cotton genome evolutionary study. The *CICR* elements also useful for further analysis of investigating phenotypic differences between A genome and D genome species.

## Additional files

Additional file 1: **Figure S1.** Consensus sequences of *CICR*-LTRs in *G. arboreum*, *G. barbadense*, *G. hirsutum*. The consensus of *CICR*-LTRs in *G. arboreum*, *G. hirsutum*, *G. barbadense*, were trained from all the CICR-LTRs of three genomes respectively. (TIF 4749 kb)

Additional file 2: **Figure S2.** Distributions of *CICRs* and genes in *GhAt*. The outermost arc strip with scale (unit: Mb), represents the At chromosome. The black bands and grey regions in chromosome represent intact *CICRs* and C-Regions, respectively. The red and blue histograms represent the *CICR*-LTRs and genes distribution density, respectively, *CICR*-LTRs and genes density of *Gossypium* chromosomes in 2 Mb unit. (TIF 16162 kb)

Additional file 3: **Table S1.** Major BLASTN results of Scaf 02 with D_5_ and A_2_ genome. (DOCX 19 kb)

Additional file 4: **Table S2.** The *CICR* members in *G. arboreum, G. hirsutum* and *G. barbadense*. (XLSX 22 kb)

Additional file 5: **Table S3.** The genes inserted with *CICR*-LTR in GhAt and their homologous in GhDt. (DOCX 19 kb)

Additional file 6: **Table S4**. Distribution of *CICR*-LTR in different tetraploid genome assemblies. (XLSX 633 kb)

## Abbreviations

At: A sub genome
BAC: Bacterial artificial chromosome
CDS: Coding sequence
Dt: D sub genome
FISH: Fluorescence in situ hybridization
GO: Gene ontology
LTR: Long terminal repeat
LTR-RT: Long terminal repeat retrotransposon
Mya: Million years ago
SSR: Simple sequence repeats
TE: Transposable element
TSD: Target site duplication
