# Supplementary figures and images for: Discovery and annotation of a novel transposable element family in Gossypium
Source: BMC Plant Biol. 2018 Nov 28;18:307. doi: 10.1186/s12870-018-1519-7 (PMC6264596; doi:10.1186/s12870-018-1519-7)

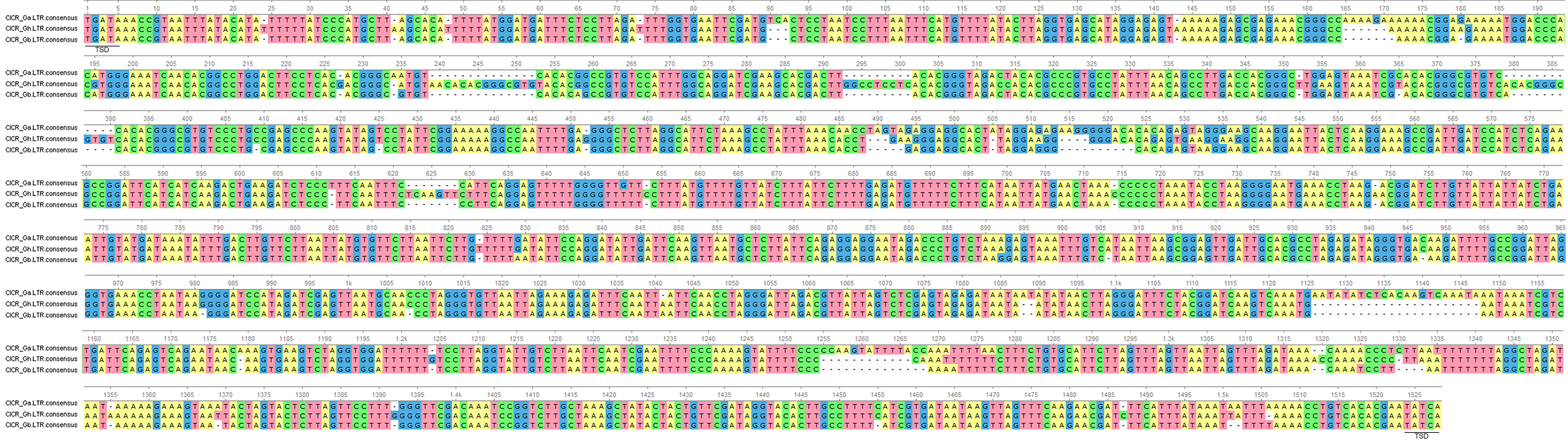

Supplement: Supplementary file 1 — Figure S1. Consensus sequences of CICR-LTRs in G. arboreum, G. barbadense, G. hirsutum. The consensus of CICR-LTRs in G. arboreum, G. hirsutum, G. barbadense, were trained from all the CICR-LTRs of three genomes respectively. (TIF 4749 kb) [file 12870_2018_1519_MOESM1_ESM.tif]

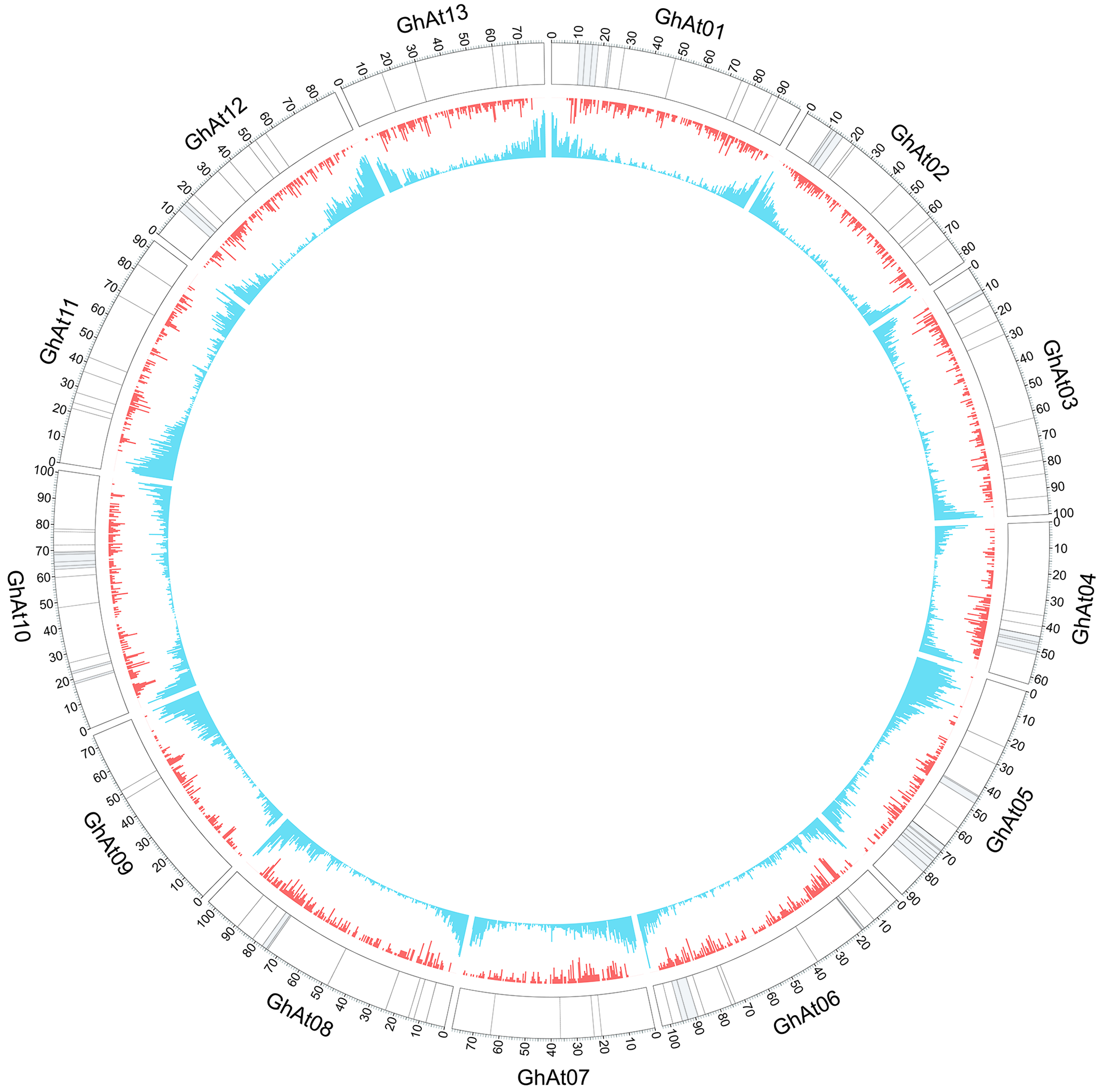

Supplement: Supplementary file 2 — Figure S2. Distributions of CICRs and genes in GhAt. The outermost arc strip with scale (unit: Mb), represents the At chromosome. The black bands and grey regions in chromosome represent intact CICRs and C-Regions, respectively. The red and blue histograms represent the CICR-LTRs and genes distribution density, respectively, CICR-LTRs and genes density of Gossypium chromosomes in 2 Mb unit. (TIF 16162 kb) [file 12870_2018_1519_MOESM2_ESM.tif]
