# Supplementary material for: Discovery and annotation of a novel transposable element family in Gossypium
Source: BMC Plant Biol. 2018 Nov 28;18:307. doi: 10.1186/s12870-018-1519-7 (PMC6264596; doi:10.1186/s12870-018-1519-7)
Supplement: Supplementary file 3 — Table S1. Major BLASTN results of Scaf 02 with D5 and A2 genome. (DOCX 19 kb) [file 12870_2018_1519_MOESM3_ESM.docx]

**Table S1.** Major BLASTN results of Scaf 02 with D_5_ and A_2_ genome

| Query | Subject | Query start | Query end | Subject start | Subject end | Align length | Identity | Repeat times |
| --- | --- | --- | --- | --- | --- | --- | --- | --- |
| Scaf02 | D_5_13 | 22772 | 26344 | 34069506 | 34073793 | 3573 bp | 97.45 | 1 |
| Scaf02 | D_5_13 | 41796 | 46057 | 34074356 | 34077911 | 4262 bp | 92.65 | 1 |
| Scaf02 | A_2_ genome | 8240 | 9613 | - | - | ~1.4 kb | >80.00 | 4931 |
