# Supplementary material for: Discovery and annotation of a novel transposable element family in Gossypium
Source: BMC Plant Biol. 2018 Nov 28;18:307. doi: 10.1186/s12870-018-1519-7 (PMC6264596; doi:10.1186/s12870-018-1519-7)
Supplement: Supplementary file 5 — Table S3. The genes inserted with CICR-LTR in GhAt and their homologous in GhDt. (DOCX 19 kb) [file 12870_2018_1519_MOESM5_ESM.docx]

**Table S3.** The genes inserted with *CICR*-LTR in *GhAt* and their homologous in *GhDt*.

| Gene ID | **Gene size (bp)** | **Exons total size (bp)** | **Protein size (aa)** | Insertion start | Insertion end | Identity |
| --- | --- | --- | --- | --- | --- | --- |
| Gh_A01G1600 | **63516** | **927** | **308** | 4530 | 5865 | 0.91 |
| Gh_D01G1858 | **2273** | **1077** | **358** |  | - |  |
| Gh_A03G1391 | **48283** | **2157** | **718** | 30149 | 31501 | 0.83 |
| Gh_D02G1851 | **47260** | **2910** | **969** | - | - |  |
| Gh_A04G0648 | **188706** | **1344** | **447** | 34701 | 36067 | 0.99 |
| Gh_D04G1112 | **1433** | **1344** | **447** | - | - |  |
| Gh_A07G0714 | **110621** | **3267** | **1088** | 49045 | 50129 | 0.8 |
| Gh_D07G0776 | **31147** | **2865** | **954** | - | - |  |
| Gh_A10G1509 | **99577** | **480** | **159** | 40820 | 42171 | 0.96 |
| Gh_D10G1753 | **579** | **447** | **159** | - | - |  |
| Gh_A11G1852 | **428268** | **2073** | **690** | 157912 | 159236 | 0.94 |
| Gh_D11G2158 | **6422** | **2022** | **673** | - | - |  |
| Gh_A11G3231 | **58351** | **2088** | **695** | 20035 | 21356 | 0.79 |
| Gh_D11G2332 | **6166** | **1977** | **658** | - | - |  |
| Gh_A12G0898 | **89793** | **5691** | **1896** | 13517 | 14882 | 0.86 |
| Gh_D12G0987 | **7009** | **5673** | **1890** | - | - |  |
| Gh_A12G1085 | **74801** | **1518** | **508** | 18714 | 20068 | 0.83 |
| Gh_D12G1208 | **53874** | **1521** | **509** | - | - |  |
| Gh_A13G0462 | **202158** | **1143** | **384** | 161664 | 163011 | 0.82 |
| Gh_D13G0686 | **114774** | **1143** | **384** | - | - |  |
| Gh_A06G1020 | **17663** | **558** | **186** | 14752 | 16117 | 0.95 |
| Gh_D06G1225 | **5529** | **1767** | **588** | - | - |  |
| Gh_A07G0841 | **82998** | **2901** | **966** | 52428 | 53784 | 0.79 |
| Gh_D07G0910 | **3356** | **1548** | **515** | - | - |  |
| Gh_A01G0536 | **96243** | **3876** | **1291** | 67143 | 68207 | 0.65 |
| Gh_D01G0550 | **3062** | **2289** | **762** | - | - |  |
| Gh_A09G0496 | **97745** | **1518** | **428** | 42536 | 43874 | 0.61 |
| Gh_D09G0503 | **72680** | **1518** | **338** | - | - |  |
| Gh_A11G2265 | **48434** | **1593** | **914** | 8034 | 9342 | 0.71 |
| Gh_D11G2573 | **4523** | **1986** | **1209** | - | - |  |
| Gh_A07G1322 | **53121** | **2745** | **914** | 10913 | 11992 | - |
| Gh_A09G0241 | **45177** | **1287** | **505** | 12579 | 13945 | - |
| Gh_A13G0875 | **100566** | **2176** | **727** | 1174 | 2526 | - |

Note: Every two adjacent genes with the same background color are homologous genes between At and Dt, while the red character represent the genes with more exons variation and green represent the new genes in GhAt.
